# Exploiting the Ref-1-APE1 node in cancer signaling and other diseases: from bench to clinic

**DOI:** 10.1038/s41698-017-0023-0

**Published:** 2017-06-08

**Authors:** Fenil Shah, Derek Logsdon, Richard A. Messmann, Jill C. Fehrenbacher, Melissa L. Fishel, Mark R. Kelley

**Affiliations:** 1 Department of Pediatrics, Herman B Wells Center for Pediatric Research, Indiana University School of Medicine, 1044 W. Walnut St., Indianapolis, IN 46202 USA; 2 Department of Pharmacology & Toxicology, Indiana University School of Medicine, 1044 W. Walnut St., Indianapolis, IN 46202 USA; 3 Apexian Pharmaceuticals, Indianapolis, IN 46204 USA

## Abstract

Reduction-oxidation factor 1-apurinic/apyrimidinic endonuclease (Ref-1/APE1) is a critical node in tumor cells, both as a redox regulator of transcription factor activation and as part of the DNA damage response. As a redox signaling protein, Ref-1/APE1 enhances the transcriptional activity of STAT3, HIF-1α, nuclear factor kappa B, and other transcription factors to promote growth, migration, and survival in tumor cells as well as inflammation and angiogenesis in the tumor microenvironment. Ref-1/APE1 is activated in a variety of cancers, including prostate, colon, pancreatic, ovarian, lung and leukemias, leading to increased aggressiveness. Transcription factors downstream of Ref-1/APE1 are key contributors to many cancers, and Ref-1/APE1 redox signaling inhibition slows growth and progression in a number of tumor types. Ref-1/APE1 inhibition is also highly effective when paired with other drugs, including standard-of-care therapies and therapies targeting pathways affected by Ref-1/APE1 redox signaling. Additionally, Ref-1/APE1 plays a role in a variety of other indications, such as retinopathy, inflammation, and neuropathy. In this review, we discuss the functional consequences of activation of the Ref-1/APE1 node in cancer and other diseases, as well as potential therapies targeting Ref-1/APE1 and related pathways in relevant diseases. APX3330, a novel oral anticancer agent and the first drug to target Ref-1/APE1 for cancer is entering clinical trials and will be explored in various cancers and other diseases bringing bench discoveries to the clinic.

## Overview of Ref-1/APE1 and its role as a cellular signaling node

Reduction-oxidation (redox) factor 1- apurinic/apyrimidinic endonuclease (Ref-1/APE1) was originally identified as an endonuclease that plays a key role in the base excision repair (BER) pathway’s repair of oxidative and alkylating damage.^1–3^ Later Ref-1/APE1 was recognized as a redox signaling protein that modulates the activity of certain transcription factors.^4, 5^ Since then, additional functions of Ref-1/APE1 have been uncovered.^6–10^ Ref-1/APE1’s duality and pivotal positions in repair and redox activities make it a unique target for therapeutic modulation.

Ref-1/APE1 endonuclease activity is vital to the DNA damage response in all cells, making Ref-1/APE1 a crucial factor in cellular function and survival.^2, 3, 11^ The repair function has been conserved from *Escherichia coli* to humans; however, the redox signaling function is observed only in mammals.^12^

**Fig. 1:**
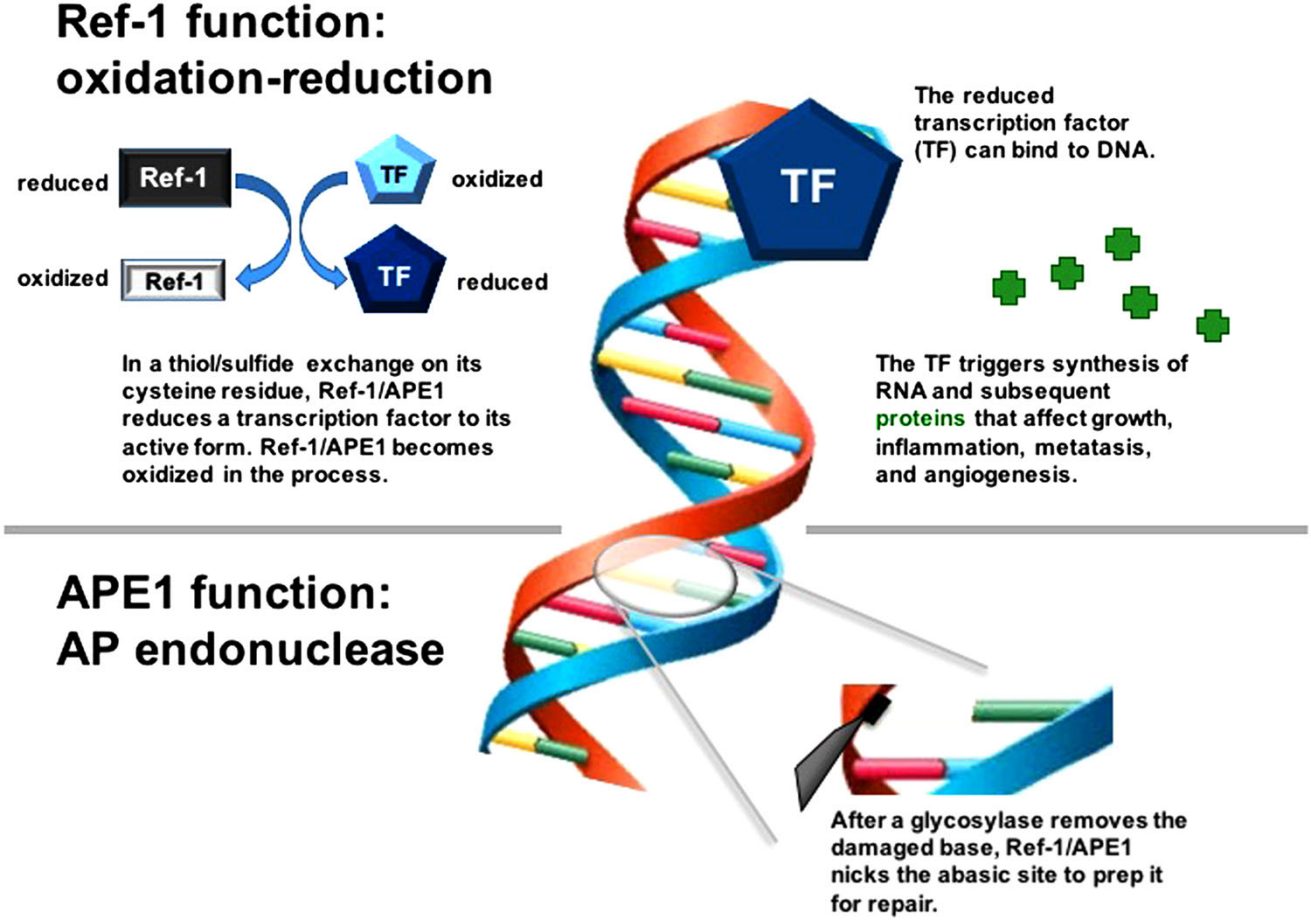
Dual functions of Ref-1/APE1. Ref-1/APE1 is a multifunctional protein involved in redox signaling and DNA repair. The redox signaling function is responsible for reduction of oxidized cysteine residues in certain transcription factors (TF’s), leading to increased transcriptional activity and upregulation of genes involved in cell growth, inflammation, angiogenesis, and other cellular functions. The DNA repair function is responsible for the endonuclease activity in base excision repair, cutting the phosphodiester backbone of DNA at abasic sites created by glycosylases. These cuts allow the abasic sites to be replaced with appropriate nucleotide bases, completing the DNA base excision repair process

Ref-1/APE1 redox signaling affects numerous transcription factors including STAT3, HIF-1α, nuclear factor kappa B (NF-κB), AP-1, p53, and a few others.^13–19^ Ref-1/APE1 redox signaling is a highly regulated process that reduces oxidized cysteine residues in specific transcription factors as part of their transactivation^4, 5, 13–24^ (Fig. 1, Table 1). Ref-1/APE1 expression is increased in many tumor types, and that change is associated with increased growth, migration, and drug resistance in tumor cells as well as decreased patient survival.^2, 3, 14, 21, 25, 26^

**Table 1:** Redox-sensitive cysteine residues in transcription factors

Transcription Factor	Redox-sensitive cysteine	Domain	Reference
STAT3	C418, C426, C468	DNA binding	^23^
HIF1α	C824	C-terminal transactivation	^24^
p50 (NFκB)	C62	DNA binding	^15^
Jun (AP-1)	C269	DNA binding	^4^
Fos (AP-1)	C154	DNA binding	^4^
p53	C275, C277	DNA binding	^16^

**Fig. 2:**
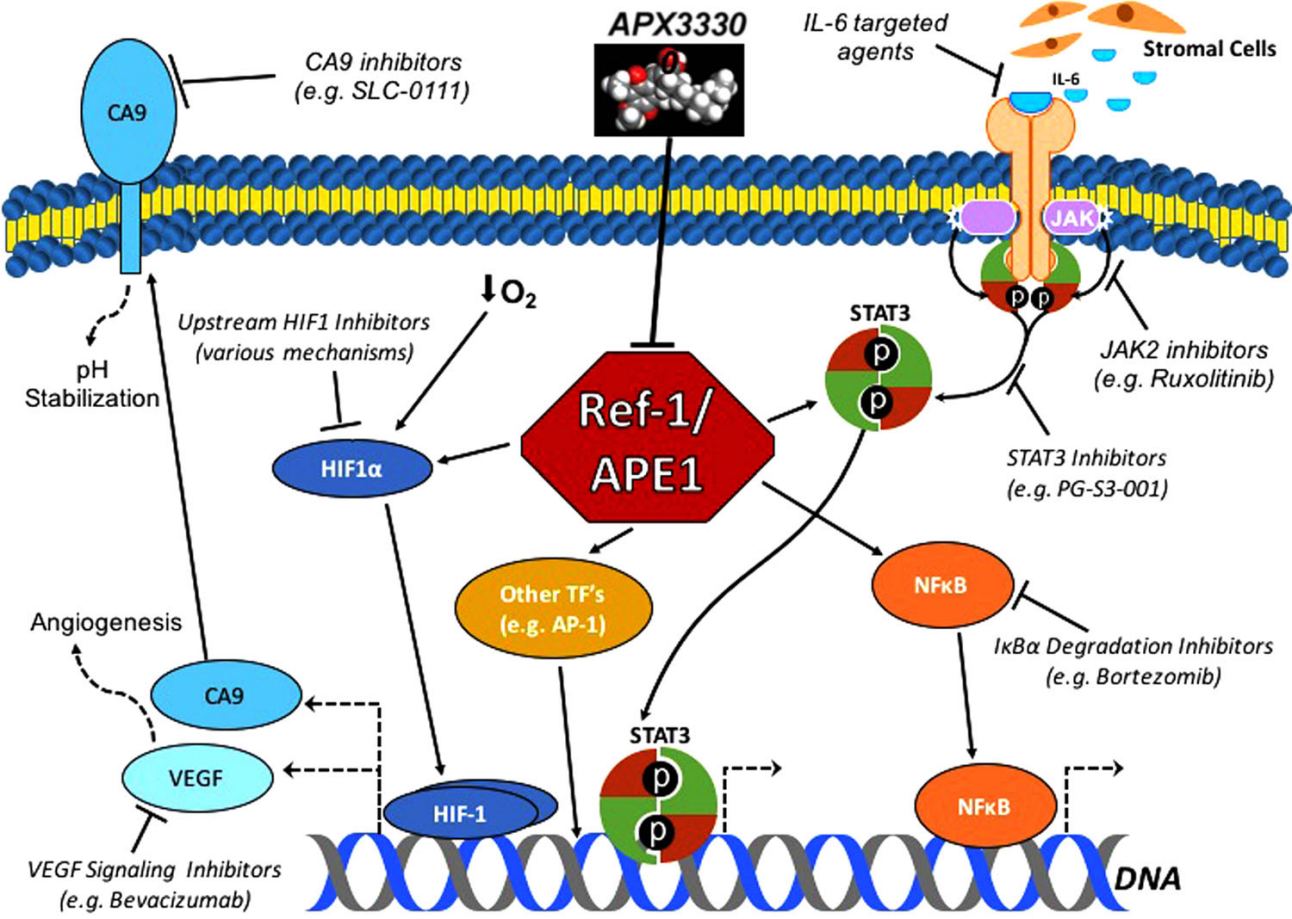
Potential inhibitors of the Ref-1/APE1 signaling node and related pathways in tumor cells. Ref-1/APE1 redox signaling promotes the transactivation of transcription factors such as STAT3, HIF-1α, and NF-κB. Inhibiting Ref-1/APE1 with APX3330 decreases the expression of downstream genes, leading to tumor cell growth arrest and/or death. Additionally, other methods for inhibiting the signaling pathways affected by Ref-1/APE1, as well as the enzymes that are upregulated by these pathways, have been shown to enhance the cytotoxic and cytostatic effects of Ref-1/APE1 inhibition

Because of the pathways it affects, Ref-1/APE1 is seen as a critical node in tumor signaling (Fig. 2) and thus is a prime target for anticancer therapy.^2, 3, 19, 21^ However, teasing apart Ref-1/APE1’s activities to create a specific inhibitor that targets only its endonuclease or redox function is challenging. This has been accomplished with the compound APX3330 (formerly called E3330), which is a specific Ref-1/APE1 redox inhibitor. APX3330 has been extensively characterized as a direct, highly selective inhibitor of Ref-1/APE1 redox activity that does not affect the protein’s endonuclease activity in tumors (Section IV; Fig. 6).^13, 17, 21, 22, 27–29^ Treatment with APX3330 slows tumor growth and progression, with limited toxicity, in both in vitro and in vivo models.^13, 18, 30, 31^ APX3330 is entering clinical trials in mid-2017 and is discussed in Section V of this review.

A number of compounds isolated from natural sources have been proposed as Ref-1/APE1 redox signaling inhibitors, but none have been shown to directly or specifically inhibit Ref-1/APE1 redox signaling.^2, 32–35^ An example of these natural compounds, resveratrol, is typical of the other compounds; its in vivo efficacy is sporadic at best due to widely varying bioavailability and low molecular specificity.^36–41^ Another presumed natural Ref-1/APE1 redox inhibitor, curcumin, has been established as a promiscuous compound, interacting with a variety of molecules to give false positive results in numerous biological assays.^41–43^ These are not specific or viable Ref-1/APE1 redox inhibitors.

Years of research for specific inhibitors of Ref-1/APE1 endonuclease activity similarly have yielded very limited results. An indirect, non-specific inhibitor, methoxyamine, a compound used as a reagent in preparation of 0-methyl oximes, also binds to abasic sites as well as free aldehydes in cells. However, its success in clinical trials has been weak, at best. Direct Ref-1/APE1 endonuclease inhibitors are still in early preclinical stages of development, so their clinical utility is yet to be determined [reviewed in refs. 2, 3]. No direct, Ref-1/APE1-specific endonuclease DNA repair activity inhibitors have moved beyond the hit-to-lead stage.^2, 3, 34^

### Targets of Ref-1/APE1 redox signaling and control

To understand Ref-1/APE1’s therapeutic potential, this section reviews its transcriptional targets, their normal and cancer-induced functions, any inhibitors in development, and Ref-1/APE1’s activities on each protein.

### STAT3

#### Normal and cancer-induced functions

Signal transducer and activator of transcription-3 (STAT3) drives the transcription of cell growth and survival genes in a variety of cell types. In response to cytokines and growth factors, Janus Kinase (JAK) phosphorylates STAT3, causing STAT3 to dimerize and translocate.^44–46^ Ref-1/APE1’s redox function directly regulates STAT3 DNA binding and transcriptional activity,^13, 44–46^ modulating STAT3’s action as a transcription activator.

STAT3 signaling promotes inflammation. Along with other molecules that drive inflammation and tumor growth/spread, STAT3 contributes to tumor progression by upregulating cytokines, growth factors, and matrix metalloproteases.^44, 47–50^ In fact, STAT3 activation is common in tumor tissue, leading to increased growth and invasiveness via transcription of genes involved in mitosis, cell motility, epithelial–mesenchymal transition, extracellular matrix remodeling, and other activities.^48, 51–56^ Hence, both STAT3 signaling and various molecules under STAT3 transcriptional control have been explored as therapeutic targets in cancer.^57–60^

#### Inhibitors in development

Attempts to develop specific, direct STAT3 inhibitors for clinical purposes have thus far fallen short.^61–63^ The newest lead compound for STAT3 Src homology 2 (SH2) domain inhibition is PG-S3-001, which has apparent selectivity (per a KINOME*scan*) and efficacy in both in vitro and in vivo models.^64^ Other compounds that can inhibit STAT3’s SH2 domain or DNA binding domain have poor specificity for a variety of reasons (reviewed in^62^).

However, other approaches to STAT signaling inhibition continue to be pursued. The JAK inhibitor ruxolitinib blocks JAK signaling, which in turn inhibits STAT3 phosphorylation and activation. Ruxolitinib has been used successfully to treat myelofibrosis and polycythemia,^65–68^ but its efficacy in solid tumors has been minimal at best.^69, 70^

Anti-IL-6 therapy has the potential to treat tumors by blocking JAK/STAT signaling.^50^ Although the clinical results thus far have been mixed, this approach continues to be studied in a variety of tumor types.^71–73^ Meanwhile, the compounds OPB-31121 and OPB-51602 are being studied as STAT3 inhibitors,^74, 75^ but with some reservations. OPB-31121 downregulates JAK2 and the IL-6 receptor gp130, thereby decreasing STAT3 activation via upstream modulators.^76^ OPB-51602 has a long half-life, and the accumulation of its active metabolite discourages daily dosing. Results on various tumor types have been mixed.^77–80^

Napabucasin (BBI-608) is being explored as a cancer stem cell inhibitor due to its effects on STAT3 activation as well as on the β-Catenin and Nanog pathways.^81–83^ Still, the search continues for STAT3 inhibitors that are suitable for clinical use.

#### Ref-1/APE1 activity on STAT3

**Fig. 3:**
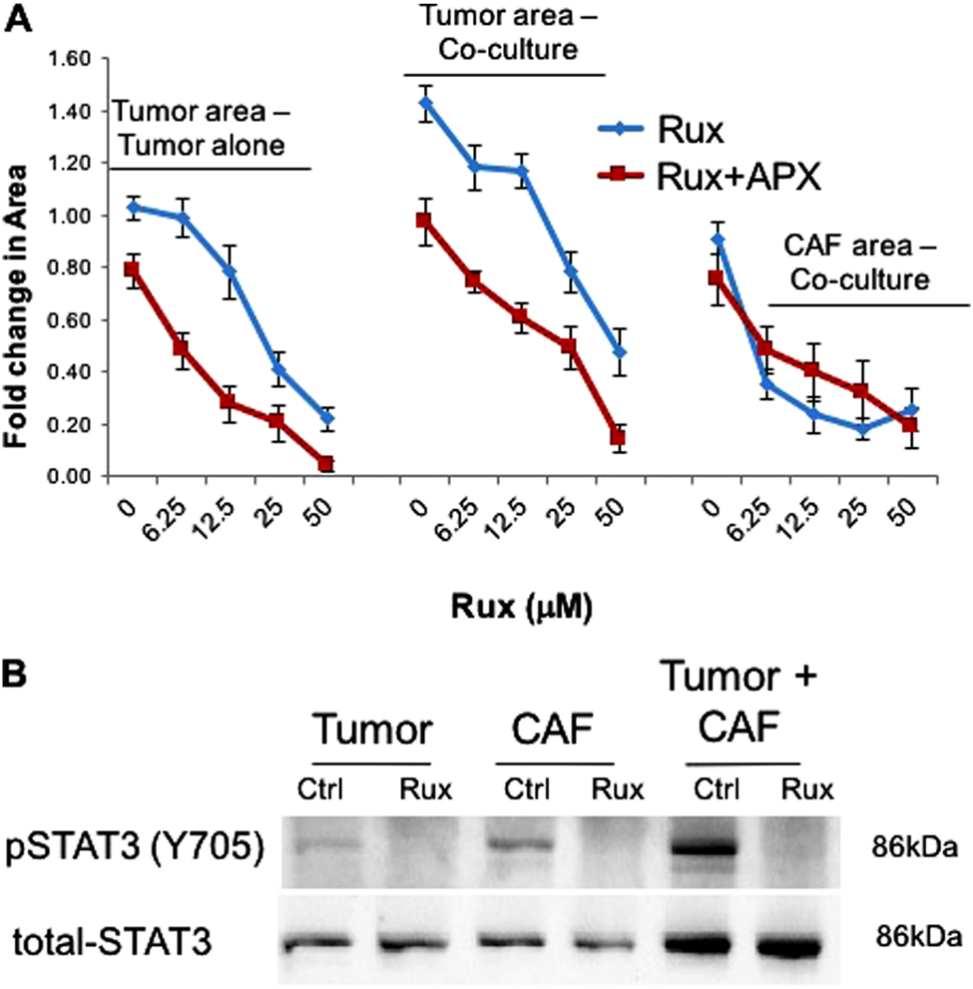
Dual-targeting of Ref-1/APE1 and Jak/STAT signaling inhibits PDAC tumor growth in a 3D co-culture model. **a** Low passage patient-derived tumor cells, Pa03C were grown in 3D cultures in the presence and absence of CAFs. Spheroids were treated with Ruxolitinib alone (*blue curve*) and in combination with APX3330 (40 μM, *red curve*), and the area of tumor (*red*) and CAF19#1 (*green*) were quantified following 12 days in culture, *n* = 4; fold change refers to comparison of drug-treated vs. media only in the tumor alone spheroids. **b** Confirmation of inhibition of STAT3 activation was done via immunoblotting for pSTAT3 Y705 residue after 4 h of Ruxolitinib treatment (12.5 μM) in the 3D assay 8–10 days post plating. Total STAT3 protein is provided as a loading control and reference for the levels of STAT3 in both cell types. Representative western blot is shown from an *n* of 3

STAT3 binding to DNA is sensitive to Ref-1/APE1 redox regulation.^13, 23, 84–86^ Thus, Ref-1/APE1 inhibition would inhibit STAT3 transcriptional activity and its downstream targets. That is seen in pancreatic cancer cells, where simultaneous targeting of Ref-1/APE1 and STAT3 signaling, using an upstream JAK2 inhibitor Ruxolitinib, synergistically inhibits proliferation and migration^13^ and slows growth in 3D pancreatic cancer spheroids (Fig. 3). Additionally, inhibition of Ref-1/APE1 redox signaling decreases expression of the STAT3 target survivin,^13^ an anti-apoptotic molecule that is a potential target for cancer therapy in its own right.^59, 87, 88^

Interestingly, data from a reverse phase protein lysate microarray suggest that JAK2 expression decreases following Ref-1/APE1 knockdown (data not shown). Further experiments are needed to validate these findings and determine whether this is caused directly by loss of Ref-1/APE1 redox signaling for transcription or a compensatory mechanism that occurs in response to loss of Ref-1/APE1 DNA repair and/or redox activity. Notably, STAT3 often acts as a cofactor with other transcription factors that are also under Ref-1/APE1 redox signaling control, including HIF-1 and NF-κB.^47, 89–96^ Hence, inhibiting Ref-1/APE1 redox signaling alone or in combination with a STAT3 signaling inhibitor could potentially provide a novel approach to cancer therapeutics.

### HIF-1

#### Normal and cancer-induced functions

Hypoxic conditions in all human cells are counteracted by hypoxia inducible factor-1 alpha (HIF-1α) stabilization and subsequent transcription of genes that upregulate cell growth, migration/invasion, and survival.^97–101^ Through HIF activity, cancer cells acquire many malignant properties, including metabolic adaptation and runaway proliferation.^101^ Because Ref-1/APE1 redox signaling promotes HIF-1 transactivation,^14, 18^ the interplay between HIF-1 and Ref-1/APE1 presents vast opportunity for therapeutic manipulation.

In normoxic conditions, HIF-1α is rapidly degraded following proline hydroxylation and von Hippel–Lindau protein-mediated ubiquitination. But, as oxygen levels decrease, stable HIF-1α levels increase, forming dimers with constitutively expressed HIF-1β (also called aryl hydrocarbon receptor nuclear translocator, ARNT) and binding to hypoxia-response elements in DNA.^100, 102–104^

#### Inhibitors in development

A drug that inhibits HIF-2α dimerization with ARNT (without affecting HIF-1α) is currently in development.^105^ But direct, HIF-1-specific inhibition is not possible yet, despite active interest in targeting HIF-1 signaling in cancer.^103, 106, 107^

Several indirect methods for targeting HIF-1α signaling exist, including mTOR inhibition to prevent HIF-1α synthesis, histone deacetylase inhibition to decrease HIF-driven transcription, and unfolded protein response inhibition to decrease transcription that is dependent on HIF cofactors such as XBP1.^103, 108–112^

Notably, FTY720 (fingolimod) has been shown to prevent the stabilization of both HIF-1α isoforms via sphingosine 1-phosphate pathway inhibition. In cell studies and a xenograft model, this led to improved tumor cell killing and vascular remodeling when used in combination with other therapies such as gemcitabine and rapamycin.^113, 114^ Similarly, the compound YC-1 downregulated HIF-1α in a MAPK-signaling-dependent manner^115^ and inhibited HIF-driven transcription via stimulation of factor inhibiting HIF.^116^

#### Ref-1/APE1 activity on HIF-1

HIF-1 transcriptional activity is under Ref-1/APE1 redox control. Under hypoxic conditions, Ref-1/APE1 inhibition decreases the expression of HIF-1-induced genes and decreases cell viability.^14, 18, 24^ Interestingly, HIF-1 cooperates with STAT3 to promote transcription of tumor-promoting factors,^89–91^ so Ref-1/APE1 redox signaling inhibition has the potential to block two transcriptional drivers at once.

Targeting hypoxic cells to inhibit tumor-promoting pathways affected by HIF-mediated transcription is already well established. For instance, VEGF inhibitors such as bevacizumab are used to block tumor-associated angiogenic remodeling; however, acquired resistance is common with those drugs.^117–120^

A major subset of the research into HIF-1 transcriptional targets focuses on pH regulatory systems in hypoxic tumor cells.^121–124^ Carbonic anhydrase IX (CA9) is a transmembrane protein that responds to hypoxia by regulating intracellular pH. CA9 promotes cell survival and invasiveness by acidifying the tumor microenvironment (TME).^124–127^ Because CA9 expression is dependent on HIF-1 activity, it is generally not detectable in normal tissue. In contrast, elevated CA9 expression in cancer tissue is thought to indicate locally advanced tumors with hypoxic regions and a poor chance of treatment response. This positions CA9 as a potential therapeutic target in a variety of tumors.^121, 122, 125, 126, 128, 129^ A number of CA9 inhibitors are under development; SLC-0111 is currently in clinical trials.^129–131^

Similarly, bicarbonate transporter inhibition was recently shown to kill hypoxic tumor cells via intracellular acidification; this effect is enhanced with CA9 knockdown.^132^ Importantly, hypoxia-induced CA9 expression decreases with Ref-1/APE1 inhibition. Thus, inhibition of Ref-1/APE1 potentiates the cytotoxic effects of CA9 inhibition in pancreatic cancer cells under hypoxic conditions by acidifying the intracellular environment.^14^

Further work is needed to explore the effects of Ref-1/APE1 redox signaling inhibition on hypoxia signaling, both alone and in combination with inhibitors of HIF, its cofactors (including STAT3), and/or the enzymes upregulated by HIF-mediated transcription.

### Nuclear factor kappa B

#### Normal and cancer-induced functions

NF-κB is a transcription factor involved in cellular responses to extracellular signals.^133–140^ NF-κB is constitutively inhibited by NF-κB Inhibitor α (IκBα), which is targeted for degradation following phosphorylation by IκB kinase (IKK). That action frees NF-κB to promote the transcription of genes involved in cell proliferation, migration, invasion, and survival.^133, 140–144^ NF-κB signaling contributes to inflammatory responses, which are a major aspect of cancer progression.^49, 133, 135, 145–147^

#### Inhibitors in development

Although specific inhibitors of NF-κB signaling previously were a hot topic in cancer research, little activity has occurred in this field recently.^148–150^ Its ubiquitous nature has far-reaching, unintended effects on healthy cells when direct inhibition is attempted.^147, 148^ In clinical settings, NF-κB signaling is inhibited indirectly by blocking the degradation of IκBα with a proteasome inhibitor such as bortezomib.^149–151^

#### Ref-1/APE1 activity on NF-κB

NF-κB DNA binding requires Ref-1/APE1 redox activity, and inhibition of Ref-1/APE1 redox signaling decreases NF-κB-mediated transcription. This indicates that NF-κB signaling is yet another cancer-associated pathway that relies on Ref-1/APE1.^15, 18^ Moreover, crosstalk between the STAT3 and NF-κB signaling pathways is well established, involving both feed-forward transcription of activating factors and STAT3/NF-κB complexes mediating co-transcription of tumor-promoting genes.^47, 92–96^ This provides further support for Ref-1/APE1 being a master regulator of both enzymes, a pivotal signaling node, and a worthy therapeutic target.

#### Other transcription factors

AP-1 is an as-yet undruggable transcription factor. Comprising Fos and Jun, AP-1’s dimerization drives transcription of proto-oncogenes, making AP-1 an “early response” transcription factor for carcinogenesis.^152–154^ AP-1-mediated transcription depends on Ref-1/APE1-mediated reduction of cysteine residues in the DNA-binding regions of both Fos and Jun.^4, 5, 155^

Ref-1/APE1 regulates both the transcriptional activity and DNA repair signaling activity of the tumor suppressor p53.^16, 156, 157^ Additionally, Ref-1/APE1’s redox function negatively regulates transcription factor nuclear factor erythroid-related factor 2 (NRF2).^17^ NRF2’s cytoprotective functions include regulation of oxidative stress; however, NRF2 also upregulates HMOX-1, a pro-tumorigenic gene responsible for treatment resistance.^158^ Combined inhibition of HMOX-1 and Ref-1/APE1’s redox activity has been shown to synergistically kill pancreatic cancer cells.^17^

Other tumor-promoting transcription factors are also under Ref-1/APE1’s redox control, including ATF/CREB, Myb, and Pax-5/8 (reviewed in ref. 19), indicating that Ref-1/APE1 redox signaling inhibition has the potential to block several tumorigenic pathways at once.

## Ref-1/APE1’s role in multiple diseases

### Cancers

**Fig. 4:**
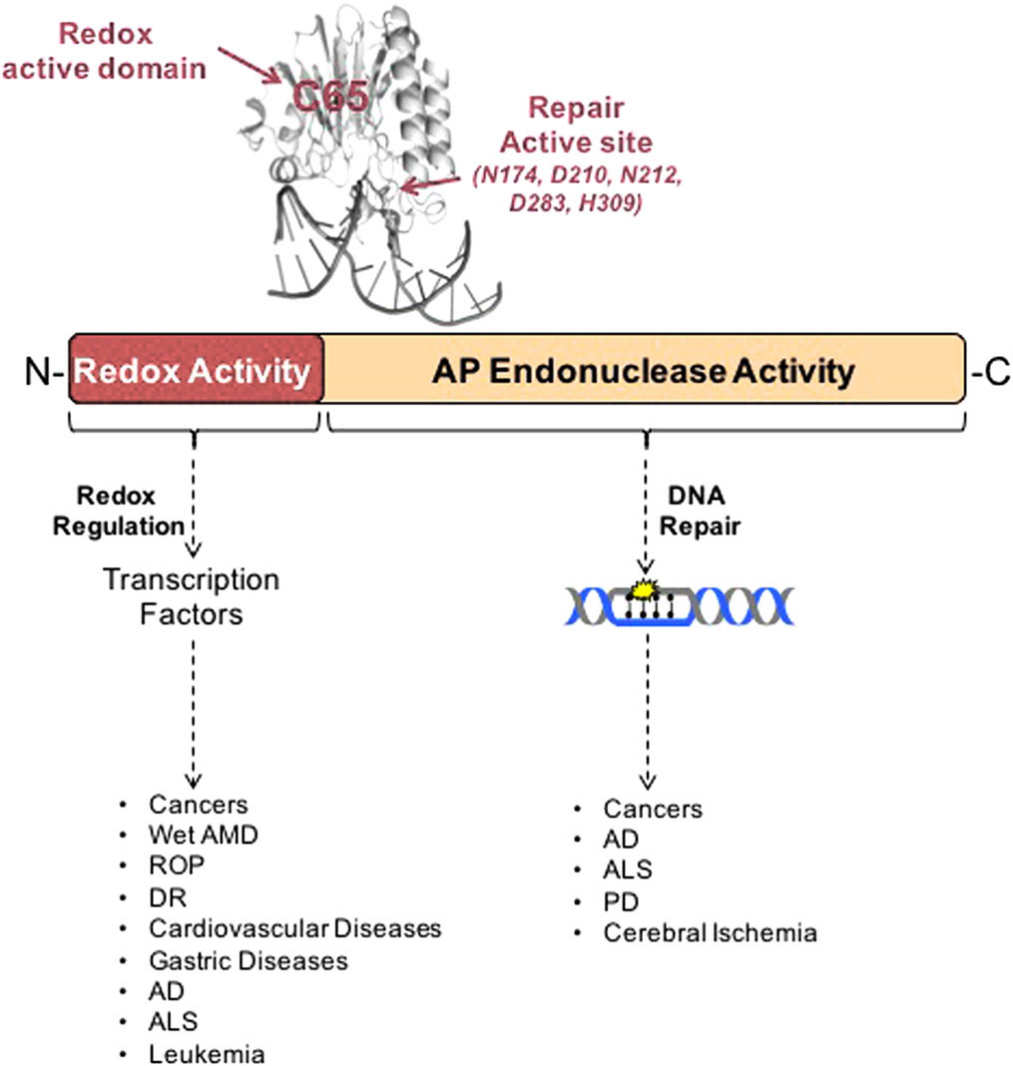
Ref-1/APE1 in human diseases. Due to its multi-functional nature, Ref-1/APE1 impacts a wide range of human diseases. Altered expression of Ref-1/APE1 affects its regulation of multiple transcriptional factors, leading to various cancers, retinal, cardiovascular, gastric and neurodegenerative diseases. Similarly, modified Ref-1/APE1 DNA repair function affects progression of different cancers and neurodegenerative diseases.^277^

The multi-functional nature of Ref-1/APE1 alludes to its expansive roles in disease, particularly cancers. Ref-1/APE1 is upregulated in many cancers (Table 2, Fig. 4). This increase is frequently associated with tumorigenesis, cancer aggressiveness, increased angiogenesis, radiotherapeutic and chemotherapeutic resistance, and overall poor prognosis.^21, 159–163^ This makes Ref-1/APE1 and the transcription factors it regulates prime targets for anticancer therapies.

**Table 2:** Tumor tissues/cells with increased Ref-1 expression

Cancer with increased Ref-1 expression	Reference
Prostate	^164^
Pancreatic	^25^
Cervical	^279^
Ovarian	^186, 191^
Osteosarcoma	^280^
Germ cell tumor	^281^
Colon/colorectal	^176, 179^
Bladder	^282^
Head and neck	^163^
Gastric/gastro-esophageal	^159, 191^
Neuroectodermal tumors	^162^
Rhabdomyosarcomas	^283^
Pancreaticobiliary	^191^
Adult gliomas	^284^
Non-small cell lung	^193^
Hepatocellular	^160^
Multiple myeloma	^285^
Esophageal	^286^
Breast	^287^
Pediatric ependymoma	^288^
Melanoma	^35^

#### Prostate cancer

One of the most widely studied cancers that exhibits Ref-1/APE1 overexpression is prostate cancer. Overexpression is seen immunohistologically as a higher percentage of cells staining positive for Ref-1/APE1 in the cytoplasm and an increased intensity of Ref-1/APE1 nuclear staining.^164^

One of the main targets of Ref-1/APE1 redox signaling in prostate cancer is STAT3, which is constitutively active in prostate cancer. STAT3 inhibition suppresses prostate cancer cell growth.^165–167^ Conversely, STAT3 activation negatively affects overall survival rates^168^ and shortens relapse-free survival (RFS).^169^ Ninety five percent of metastatic samples taken from patients who died of castration-resistant prostate cancer were positive for pSTAT3, with the highest expression seen in bone metastases samples.^170^ Collectively, this supports the crucial role of pSTAT3 in prostate cancer aggressiveness and progression.

A downstream target of STAT3 is survivin; its increased expression is also associated with prostate cancer aggressiveness.^171^ mRNA expression levels of survivin in prostate biopsy tissues show significantly higher survivin expression in cancerous tissue, which correlates with higher-grade cancer and aggressive phenotypes.^172^ siRNA knockdown of survivin in prostate cancer cell lines reduces cell proliferation^172^ and increases chemosensitivity to the apoptosis-inducing agent cisplatin.^173^ The effects of decreased survivin expression extend in vivo. Mice injected subcutaneously with siRNA survivin knockdown cells exhibit significantly smaller tumors compared with controls.^173^

Interestingly, Ref-1/APE1 redox-specific inhibitors APX3330 and APX2009 decreased survivin mRNA and protein levels in prostate cancer cells by affecting NF-κB activity. These inhibitors also reduced cell proliferation. In vivo, APX2009 reduced survivin protein levels and cell proliferation.^174^

Based on the evidence, both STAT3 and survivin present as prime targets for anti-prostate cancer therapies. However, to date they have been only moderately successful as single-agent therapies. Therefore, the potential combination of inhibiting both Ref-1/APE1 redox function and STAT3/survivin provides an avenue of targeting both the overarching regulator and downstream effector of an anti-apoptotic pathway integral to prostate cancer.

#### Colon cancer

Colon cancer, the second leading cause of cancer related death in the U.S.,^175^ exhibits increased levels of cytoplasmic Ref-1/APE1.^176^ In liver tumor tissue of metastasized colorectal cancer, increased Ref-1/APE1 expression corresponds to poor patient outcome.^177^ In colony-forming assays, siRNA Ref-1/APE1 knockdown significantly increases the sensitivity of colon cancer cells to ionizing radiation (IR). Furthermore, in vivo subcutaneous xenografts also show reduced tumor growth and radiosensitization following intratumoral Ref-1/APE1 siRNA treatment.^178^

The importance of Ref-1/APE1 redox signaling in colon cancer is highlighted by the effects that the Ref-1/APE1 redox inhibitor APX3330 has on colon cancer stem cells (CCSCs). APX3330 significantly reduces CCSC growth in vitro and enhances the cytotoxicity of 5-fluorouracil (5-FU), an anti-metabolite chemotherapeutic. In xenograft mice injected subcutaneously with CCSCs, intratumoral administration of APX3330 increases tumor response to 5-FU delivered intraperitoneally.^179^ This indicates that APX3330 could potentiate other colon cancer treatments by inhibiting Ref-1/APE1’s crucial redox activity.

#### Pancreatic cancer

One of the most lethal cancers, pancreatic ductal adenocarcinoma (PDAC) has a 5-year mortality rate of ~92% and is characterized by a complex TME, heterogeneity within the tumor, extreme hypoxia, and an inherent ability to metastasize.^180–183^ The regulation of HIF-1α, STAT3, AP-1, and NF-κB by Ref-1/APE1 indicates that inhibition of Ref-1/APE1 redox signaling has potential clinical utility in PDAC.

Ref-1/APE1 levels are elevated in human pancreatic cancer tissue and peri-pancreatic metastasis, and decreasing the expression of Ref-1/APE1 via siRNA in pancreatic cancer cells results in apoptosis, cell cycle arrest, and a decrease in proliferative capacity.^25^ Inhibition of Ref-1/APE1 via APX3330 inhibits the proliferation and adhesion of pancreatic cancer cell lines, arrests cell cycle progression, and decreases the transcriptional activation of major transcription factors known to be important in pancreatic cancer progression, survival, metastasis, and response to hypoxia (HIF-1α, NF-κB, STAT3 & AP-1), in addition to blocking tumor growth in vivo in patient-derived models.^13, 18^

Within the TME, there are several cell types that contribute to the growth and spread of pancreatic cancer: endothelial cells, pericytes, tumor-associated macrophages, lymphocytes, activated pancreatic stellate cells, and other cancer-associated fibroblasts (CAFs).^182^ The role of Ref-1/APE1 in the TME is still under investigation, but there is published evidence^184^ that Ref-1/APE1 can block endothelial cell function and suppress tumor-associated macrophages. Preclinical studies show that the Ref-1/APE1 inhibitor APX3330 inhibits growth of pancreatic cancer-associated endothelial and endothelial progenitor cells.^185^ Additional studies demonstrate that APX3330 can reduce tumor endothelial VEGF secretion, blocking a potentially critical angiogenic ligand-receptor interaction in the TME.^185^ Based on these data and the regulation of key transcription factors implicated in PDAC, PDAC is one of the indications for APX3330 in clinical trials.

#### Ovarian cancer

Ref-1/APE1 expression in ovarian cancer has been studied widely. Ref-1/APE1 expression is increased in malignant patient tissue samples, but studies vary as to the location of this increase.

Some studies show increased expression primarily in the cytoplasm.^186, 187^ In those studies, cytoplasmic localization of Ref-1/APE1 correlates with ovarian tumor progression. Additionally, patients with advanced (stage III/IV) cancer have significantly higher Ref-1/APE1 expression and lower overall survival rates than stage I/II patients.^188^ Patients with increased cytoplasmic Ref-1/APE1 are also more resistant to platinating chemotherapeutics.^189^ A different study observed increased Ref-1/APE1 nuclear expression, again with greater increases in stage III/IV compared to stage I/II patients.^190^ In other studies, both nuclear and cytoplasmic Ref-1/APE1 expression were increased^189, 191^—but no correlation was observed between Ref-1/APE1 expression and the cancer stage.^191^ Collectively, these studies highlight that, while Ref-1/APE1 expression clearly plays a role in ovarian cancer, the heterogeneity of tissue samples makes it hard to discern the roles of Ref-1/APE1 nuclear vs. cytoplasmic localization.

However, reduced expression of Ref-1/APE1 has a clear effect on ovarian cancer cells. Ref-1/APE1 knockdown in A2780 (nuclear Ref-1/APE1) and CP70 (cytoplasmic Ref-1/APE1) cells sensitizes both to cisplatin.^189^ In SKOV3 and A2780 cells, Ref-1/APE1 siRNA significantly reduces cell proliferation, colony formation, migration and invasion.^187, 192^ Similarly, Ref-1/APE1 siRNA treatment of SKOV-3x ovarian cells significantly reduces their growth; the same occurs with APX3330 redox inhibition.^11, 29^ Ref-1/APE1 siRNA cells implanted subcutaneously in mice show markedly reduced growth compared to control tumors: a 3.2-fold increase in tumor-doubling time (from 5 to more than 15 days). The tumors also exhibit reduced glucose metabolism.^11^ Taken together, a strong case can be made for targeting Ref-1/APE1 in ovarian cancer as a means to inhibit growth as well as enhance activity of other anticancer drugs.

#### Non-small cell lung carcinoma

Ref-1/APE1 has long been considered a prognostic marker in non-small-cell lung carcinoma (NSCLC), as Ref-1/APE1 protein levels are upregulated in patient tumor samples.^193–195^ Nuclear Ref-1/APE1 expression in tissue samples presents better survival chances for patients.^196^ Cytoplasmic Ref-1/APE1 and mRNA expression correlate strongly with poor patient survival and shorter RFS.^197–199^ Both immunohistochemistry and immunoblotting show increased cytoplasmic and reduced nuclear Ref-1/APE1 expression in patients with a recurrence of stage I NSCLC.^199, 200^ Post-treatment serum Ref-1/APE1 levels are inversely associated with overall survival.^81, 201^

Ref-1/APE1 affects platinum-based drugs commonly used in NSCLC. An increase in Ref-1/APE1 expression in NSCLC confers resistance to cisplatin treatment, while Ref-1/APE1 siRNA knockdown in A549 cancer cells significantly enhances cisplatin cytotoxicity.^202^ Patients with tumors not expressing Ref-1/APE1 respond better to platinum-paclitaxel therapy^203^ and cisplatin-docetaxel-gemcitabine treatment,^81^ with longer time to progression and overall survival.

Evidence exists that reducing Ref-1/APE1 increases the efficacy of other anticancer treatments in NSCLC. Decreasing Ref-1/APE1 levels in A549 cells in vitro and in vivo increases the effectiveness of photodynamic therapy.^204^ Ref-1/APE1 knockdown with shRNA enhances the anti-tumor activity of oxymatrine, an alkaloid compound that inhibits proliferation of A549 cells.^205^

Collectively, this demonstrates that Ref-1/APE1 plays a vital role in NSCLC progression, and targeting it might lead to better patient outcomes when combined with various chemotherapeutic treatments.

#### Malignant peripheral nerve sheath tumors (MPNST)

MPNST is an uncommon neural-origin cancer that can be deadly. Despite much research to date, existing chemotherapeutic agents have not been successful in MPNST treatment.^206^ Recent research implicates Ref-1/APE1 redox targets HIF-1α and particularly STAT3 in driving MPNST.

Phosphorylated STAT3 expression may indicate aggressive disease at disease onset. A tissue microarray showed STAT3 expression in primary MPNST was associated with worse disease-specific overall survival and event-free survival.^207^ In a mouse model of EGFR overexpression, both a JAK/STAT3 inhibitor and STAT3 knockdown by shRNA prevented tumor formation.^208^

In another study, inhibition of STAT3 activation in four MPNST lines resulted in decreased wound healing, cell migration, invasion, and tumor formation. It also reduced HIF-1α expression. Independent shRNA-mediated HIF-1α knockdown also decreased wound healing, cell migration, invasion, and tumor formation, showing that the STAT3/HIF-1 α signaling pathway is responsible for tumorigenesis in MPNST.^90^

Furthermore, STAT3’s downstream target survivin is amplified in MPNSTs.^209^ Survivin is highly expressed in MPNST tissue samples. Survivin knockdown via siRNA decreases cell growth, inhibits cell cycle progression and increases apoptosis. Additionally, survivin inhibitor YM155 represses MPNST xenograft growth and metastasis in vivo.^210^

The role of the STAT3-HIF-1α pathway in MPNST supports the notion of STAT3 and/or HIF-1α inhibition as a potential way to treat MPNST. Downstream markers like survivin also present as potential targets. Ref-1/APE1 regulates STAT3 as well as HIF-1α; therefore, targeting Ref-1/APE1 would inhibit multiple targets, providing hope for a viable treatment for MPNST. Additionally, the possibility of dual targeting Ref-1/APE1 and either STAT3 or HIF-1α alludes to the potential of completely eliminating a pathway that is integral to MPNST progression.

#### Leukemia

Few studies have focused on the role of Ref-1/APE1 in leukemias. To date the only published studies have concentrated on the role of Ref-1/APE1 in acute promyelocytic leukemia (APL) and its relationship to all-trans retinoic acid (ATRA, or RA) and retinoic acid receptor (RAR) transcription factors. RAR alpha binds to its DNA binding site retinoic acid response element (RARE) in a redox-dependent fashion. Studies by Fishel et al^27^ demonstrate that RAR–RARE binding is blocked through Ref-1/APE1 redox inhibition using APX3330. Additionally, the addition of APX3330 to ATRA increases apoptosis and cellular differentiation of APL cells by three-fold. These results indicate the potential of using APX3330 in combination treatment with ATRA. This could accomplish two things; first, a new treatment combination for leukemias where ATRA is used, and second, a reduction in the ATRA dose while maintaining similar or increased therapeutic effect. This latter point is important, as one should be able to avoid the toxicity of RA differentiation syndrome by being able to increase RA-induced promyeloblast differentiation, but with lower amounts of RA. Reducing the dose of RA has important clinical implications and could help to eliminate some of the undesirable side effects of this therapy.^211, 212^ RA administration can cause differentiation syndrome in 25% of patients.^213, 214^

Recent studies show Ref-1/APE1 is highly expressed in T-cell acute lymphoblastic leukemia (T-ALL). Blockade of Ref-1/APE1 by the redox-specific inhibitor APX3330 potently inhibits viability of leukemia T-cells, including primary cells, relapsed and chemotherapy-resistant cells, and cells from a mouse model of T-ALL (Ding et al, manuscript submitted). Ref-1/APE1 redox inhibition promotes leukemia cell apoptosis, which is associated with downregulation of pro-survival genes. These data demonstrate a role for Ref-1/APE1 in the regulation of multiple transcriptional programs in T-cell ALL, and suggest that disruption of Ref-1/APE1 redox function represents an efficient strategy to target leukemia T-cells, including high-risk, relapsed leukemias.

Finally, investigators studying conversion of pre-leukemic acute myeloid leukemia (AML) cells with TET2 mutations to full-blown AML have identified a significant role of Ref-1/APE1 in this process. Tet2-deficient stem cells demonstrate resistance to inflammatory challenge as revealed by a higher repopulating and engraftment potential in both primary and secondary recipients compared to wildtype controls, which, when stressed, show a remarkable decline in overall engraftment (Cai et al, manuscript submitted). This process invokes the NF-κB pathway, which Ref-1/APE1 regulates. APX3330 blocks NF-κB function, which decreases inflammation and reverses the progression from pre-AML to frank AML in mice bearing AML-associated epigenetic mutations often observed in healthy individuals with clonal hematopoiesis (Cai et al, manuscript submitted). These data suggest that APX3330 treatment could clinically benefit normal individuals carrying *TET2* mutations that show signs of clonal hematopoiesis, as well as patients with *TET2* mutations who have AML, myeloproliferative disease and myelodysplastic syndrome.

In summary, while studies on Ref-1/APE1 in leukemia trail behind research performed on solid tumors, recent investigations are uncovering a critical role of Ref-1/APE1 redox signaling and effectiveness of APX3330 in those leukemias investigated. Further work is ongoing in this area.

### Retinal diseases

Increased levels of Ref-1/APE1 are not limited to cancers (Fig. 4). Elevated Ref-1/APE1 has been implicated in age-related cataracts. Ref-1/APE1 levels are higher in the lens epithelium cells of patients vs. controls, and Ref-1/APE1 levels decrease as the opaque degree worsens.^215^

Ref-1/APE1 is highly expressed in developing murine retinas, as well as retinal pigment epithelium (RPE) cells, retinal pericytes, choroid endothelial cells (CECs) and RVECs.^216–218^ Using the Ref-1/APE1 inhibitor APX3330 shows that Ref-1/APE1 redox activity is required for RVEC proliferation, migration and angiogenesis in vitro.^29, 217^ Similarly, APX3330 treatment reduced proliferation, migration and angiogenesis in CECs in primate cells in vitro and had an additive effect when combined with bevacizumab.^219^ RPEs stressed using oxidized low-density lipoproteins were rescued from proliferation decline and senescence by APX3330.^218^

In adult human RPE cell lines, APX3330 reduced the transcriptional activity of NF-κB, a key factor associated with inflammation in angiogenesis.^218, 219^ It also blocked activation of HIF-1α and reduced the expression of its downstream target VEGF.^219^ VEGF expression via NF-κB and HIF-1α is primarily responsible for choroidal neovascularization (CNV), a characteristic of neovascular age-related macular degeneration (AMD), also known as wet AMD.

When very low density lipoprotein receptor knockout mice are treated with a single intravitreal injection of APX3330, CNV is reduced.^217^ APX3330 also shows anti-angiogenic effects in mice with laser-induced CNV.^219^

Angiogenesis is also a prime component of other retinal diseases, including retinopathy of prematurity (ROP) and diabetic retinopathy (DR). Ref-1/APE1’s redox ability to modulate angiogenesis makes it worth investigating in those diseases. Interestingly, both HIF-1α and VEGF are increased in ROP and DR. Retinal neovascularization, a marker of ROP and DR, is markedly reduced in mice with ischemic retinopathy when treated with siRNA targeting HIF-1α or VEGF.^220^

However, the difficulties in creating druggable targets for HIF-1α have already been discussed. Additionally, ocular anti-VEGF therapies are not always effective and may lead to unwanted side effects.^221^ Inhibiting the redox activity of Ref-1/APE1 may prove to be a more efficacious standalone or adjunctive treatment that can modulate HIF-1α and VEGF in retinal diseases like wet AMD, ROP and DR.

### Other diseases

Ref-1/APE1 has also been shown to play a role in several other diseases. Ref-1/APE1’s involvement in cardiovascular disease and regulation of blood pressure is illustrated by aortic coarctation-induced hypersensitive rat models showing increased Ref-1/APE1 expression levels.^222^ Furthermore, heterozygous Ref-1/APE1^+/−^ mice exhibit hypertension and diminished endothelium-dependent vasorelaxation.^223^ Ref-1/APE1 is part of the SET complex of proteins that are involved in HIV pathogenesis by inhibiting suicidal autointegration. Consequently, knocking down Ref-1/APE1 inhibits HIV infection.^224^

Ref-1/APE1 is also implicated in gastric cellular response to *Helicobacter pylori (H. pylori)* infection. Ref-1/APE1 expression levels were elevated following *H. pylori* infection in human gastric epithelial cells.^225^ *H*. *pylori* induced ROS and downstream activated genes were higher in Ref-1/APE1 deficient cells compared to control, with Ref-1/APE1 overexpression reversing these effects.^226^ Additionally, Ref-1/APE1 siRNA knockdown inhibited *H. pylori* and TNF-α-induced AP-1 and NF-κB DNA binding, as well as IL-8 mRNA expression and protein secretion in gastric epithelial cells. Collectively, that implicates Ref-1/APE1 in gastric inflammatory disorders.^227, 228^

Another area of particular interest is neurodegenerative disease (ND). NDs such as Alzheimer’s disease (AD), Parkinson’s disease (PD), amyotrophic lateral sclerosis (ALS) and cerebral ischemia are all affected by Ref-1/APE1 dysfunction.

Ref-1/APE1 protein levels are elevated in nuclear extracts from the midfrontal cortex^229^ and cerebral cortex^230^ of AD patients compared to controls, with Ref-1/APE1 redox activity seen as a compensatory mechanism for increased oxidative stress. However, reduced Ref-1/APE1 endonuclease activity is seen in peripheral blood mononuclear cells of AD patients, suggesting impaired BER.^231^ This highlights the different roles that Ref-1/APE1 can have within a particular disease. Similarly, Ref-1/APE1 levels are elevated in the central nervous system of patients with ALS, a disease exhibiting elevated oxidative stress and DNA damage.^232^ In PD, loss of Ref-1/APE1 function via gene variants suggests it is a risk factor, contributing to increased oxidative stress that leads to loss of dorsal root ganglion (DRG) neurons.^233^ Ref-1/APE1 is upregulated in cells treated with rotenone^234^ and MPP+ (1-methyl-4-phenylpyridinium),^235^ both of which are used to simulate a PD model. Ref-1/APE1 upregulation protects against neuronal death in these cells.^235^

After cerebral ischemia, upregulation of Ref-1/APE1 protects hippocampal neurons from cell loss and DNA fragmentation. Conversely, transgenic rats with DNA repair-compromised Ref-1/APE1 are not protected from ischemic injury.^236^ Ref-1/APE1 conditional knockout mice exhibit larger infract volume and diminished recovery of spatial and cognitive function following cerebral ischemia.^237^

These findings highlight the wide range of diseases affected by Ref-1/APE1, indicating that it is a promising target for treating and managing numerous diseases.

## Identification of additional pathways for combinatorial drug approach

A proposed factor in the limited success of molecular therapies has been the heterogeneity found in tumor samples, especially aggressive ones such as pancreatic tumors or glioblastomas. This underscores the need for strategies that target nodal proteins capable of affecting multiple pathways, such as Ref-1/APE1.^180, 181, 238^ The evaluation of novel targets including Ref-1/APE1 and rationally designed combination therapy, including correlative biomarker research, is critical in cancer because therapeutic options for some cancer patients remain limited.

To elucidate synthetic lethal pairs of chemotherapeutic agents, two general approaches are utilized. They include rational, hypothesis-driven combinations based on the mechanism of action of the compounds as well as application of “big data” that reveal specific gene expression profiles or proteomic signatures that would render cancer cells vulnerable when used in combination. To that end, this section focuses on combination therapy that involves Ref-1/APE1 modulation that results in even greater enhancement than either agent alone; synergistic effects.

Many studies have investigated the potentiation of DNA-damaging agents in combination with Ref-1/APE1 inhibition^239–241^). Presumably the predominant mechanism of potentiation in these studies was due to blockade of Ref-1/APE1’s DNA repair function, which led to cellular inability to respond to the DNA damage caused by the chemotherapeutic agent. In this review, we focus on published studies of inhibition of Ref-1/APE1 redox function or treatment with Ref-1/APE1 siRNA (Table 3).

**Table 3:** Combination treatment involving Ref-1 modulation

Molecular target/therapeutic agent paired with Ref-1	Pathways affected	Model system	Reference
Doxorubicin	Hypoxia/ABC transporter expression	Colon Cancer	^243^
STAT3	Viability/Migration	PDAC	^13^
Avastin	Angiogenesis	Retinopathy	
DNA damage (cisplatin) / Bcl-2 inhibitor	Proliferation/Migration/Apoptosis	NSCLC	^202, 244^
Platinating agents (cisplatin/oxaliplatin/carboplatin)	Attenuation of vasodilatation of sensory neurons	Chemotherapy-induced neuropathy	^254^
CA9	Hypoxia	PDAC	^14^
WNT/β-catenin	ROS/Proliferation	PDAC	^246^
Endostatin	Angiogenesis	Osteosarcoma	^242^
5-FU	Proliferation/Tumor growth	Colon Cancer	^179^
Retinoic acid	Differentiation	Promyelocytic leukemia	^27^
Photodynamic therapy (PDT)	Proliferation/TFAM (transcription factor A, mitochondria) binding	NSCLC	^289^

### Pairing therapeutic agents with Ref-1/APE1 based on its known functions in cancer cells

First, a hypothesis-driven approach is used to test chemotherapeutic agents in combination with Ref-1/APE1 inhibitors to screen for synthetic lethality. This approach involves simultaneously impinging upon Ref-1/APE1 signaling in conjunction with another key pathway that interacts with or depends upon Ref-1/APE1 function for tumor cell survival. The combination of the two should create a synthetic lethality, dramatically enhancing cell death compared to their effect when administered alone.

Using this approach, our group discovered that impinging upon STAT3 signaling in combination with Ref-1/APE1 signaling dramatically affects the viability and migratory ability of pancreatic cancer cell lines (Fig. 3 and ref. 13).

Several studies of different cancers support the notion that combination therapy involving inhibition of Ref-1/APE1 in tumor-promoting processes such as hypoxia or angiogenesis is efficacious. In an osteosarcoma model characterized by hypoxia and angiogenesis, inhibition of Ref-1/APE1 in combination with endostatin demonstrates in vivo efficacy with decreases in VEGF expression and microvessel density.^242^

Another synthetic lethal pair involving Ref-1/APE1 and hypoxia is the combination of Ref-1/APE1 inhibition with inhibition of the HIF-1α target CA9.^14^ Using pancreatic 3D co-culture models, tumor spheroid area is reduced after dual targeting with Ref-1/APE1 and CA9 (Fig. 2). The mechanism of enhancement is believed to be due to an increase in pH and blockade of the tumor’s ability to adapt to hypoxic conditions perpetuated through simultaneous CA9 and Ref-1/APE1 blockade.

Finally, studies comparing doxorubicin-sensitive vs. doxorubicin-resistant colon cancer cells demonstrate that hypoxia enhances the expression of Pgp (P-glycoprotein) and BCRP (breast cancer resistance protein)—and that the addition of APX3330 to doxorubicin under hypoxic conditions can attenuate HIF activity significantly, blocking the upregulation of Pgp and BCRP. This decrease in Pgp and BCRP expression may play a role in the observed increase in doxorubicin accumulation, especially in the parental cells.^243^ The results suggest that, when blockade of Ref-1/APE1’s redox function blockades HIF signaling, colon cancer cells’ response to doxorubicin may be enhanced. A caveat to these studies is that hypoxia was chemically induced using cobalt chloride rather than lowering oxygen levels in the cells, and only Pgp seemed to be regulated at the transcriptional level by hypoxia and doxorubicin treatment. This would suggest that additional mechanisms of drug resistance are at play.

A recent study by Ren et al.^244^ sought to sensitize NSCLC cell lines to cisplatin by sequential use of AT-101 (gossypol) with cisplatin. AT-101 exerts its anti-tumor effects in many ways: it is a BH3-mimetic and also has been shown to inhibit Ref-1/APE1’s DNA repair and redox activities. Blockade of the anti-apoptotic proteins Bcl-2 and Bcl-XL through Ref-1/APE1’s redox inhibition of STAT3 activity contributes to the enhanced cell killing and tumor growth seen in this combination.^244^ Furthermore, in NSCLC cell line A549, siRNA inhibition of Ref-1/APE1 expression significantly sensitizes A549 cells to cisplatin and increased cell apoptosis.^202^ Both of these studies point to Ref-1/APE1 function as critical in the cells’ response to cisplatin, especially in apoptosis signaling through STAT3.

In contrast, a recent study in breast cancer cell lines that were exposed to cisplatin in combination with inhibitors of either Ref-1/APE1 repair or Ref-1/APE1 redox activity, cisplatin resistance increased.^245^ The authors conjecture that a concurrent downregulation of mismatch repair proteins (MSH2, MSH6, MLH1, and ERCC1) may explain why those results differ from the other studies that demonstrate a greater response to cisplatin when Ref-1/APE1 is inhibited concurrently. In the pursuit of personalized medicine, these preclinical studies demonstrate the importance of elucidating cell-specific signaling following chemotherapy as well as the crosstalk between DNA repair pathways that occurs following DNA damaging agents. These factors will need to be considered as new treatment combinations are proposed, such as considering the addition of a Ref-1/APE1 inhibitor to a cisplatin regimen.

Finally, both Ref-1/APE1 inhibition via APX3330 and siRNA knockdown of Ref-1/APE1 upregulates β-catenin in pancreatic cancer cells. When the WNT/β-catenin inhibitor IWR-1 was paired with APX3330, enhanced cytotoxicity occurred.^246^

These examples show how seemingly “separate tracks” of cancer survival pathways can intersect, how those intersections involve Ref-1/APE1, and the exciting therapeutic possibilities that arise from those intersections.

### Mining big data to predict combination therapy involving Ref-1/APE1

A second option for uncovering new treatment options is to mine publicly available data sets such as TCGA (The Cancer Genome Atlas) and Cancer Cell Line Encyclopedia to elucidate [in silico] effective combination treatments to utilize in cancer treatment settings.^247, 248^ The goal is to accelerate the selection of likely synthetic lethal targets, particularly for aggressive cancers that have few treatment options.

For example, historically in pancreatic cancer, new targeted agents would be paired with the standard-of-care agent, gemcitabine. But adding selective inhibitors of multiple cancer-related pathways to gemcitabine either did not extend survival significantly or although statistically significant did not extend the 5-year survival rate.^249^ In today’s age of omics, “big data” can be used to predict synthetic lethality and effective drug combinations rather than a shotgun approach.^247^

A study combining transcriptional and proteomic profiling following Ref-1/APE1 knockdown in HeLa cells reveals several pathways that are differentially expressed following Ref-1/APE1 modulation. These pathways include DNA damage, mitochondrial function, and microtubule stabilization.^250^ The downregulation of DNA repair proteins following Ref-1/APE1 knockdown is another confirmation that the addition of a Ref-1/APE1 inhibitor to a DNA-damaging agent is deleterious to cancer cells.

The aforementioned study also demonstrates a downregulation in mitochondrial function. Mitochondria are emerging as important indicators of cellular disease or health following Ref-1/APE1 modulation, therefore drugs that target anti-apoptotic mechanisms may be efficacious when combined with Ref-1/APE1 inhibition. Such drugs might include Bcl-2 inhibitors or YM-155 (a survivin inhibitor). Finally, the proteomic study indicates another area in which Ref-1/APE1 inhibition may be useful as a synthetic lethality. Lack of Ref-1/APE1 expression affects microtubule stabilization proteins such as actin; impeding proper organization of the fibers. Several commonly used chemotherapeutic agents disrupt microtubule dynamics, including docetaxel, paclitaxel, and vinblastine. Therefore, those agents show promise for being able to be paired with Ref-1/APE1 inhibition.

Much is yet to be learned regarding which clinical agents will pair most effectively with Ref-1/APE1 redox or repair inhibitors; however, studies are ongoing to determine whether this approach will yield drug combinations that are synthetically lethal to cancer cells.

## Ref-1/APE1 DNA repair and CIPN; indirect impact linking through altering redox function

Chemotherapy-induced peripheral neuropathy (CIPN) is one of the most prevalent dose-limiting toxicities of anticancer therapy. Up to 90% of cancer patients experience CIPN at some point during or after anticancer treatment.^251^ Indeed, anticancer drugs used for the six most common malignancies pose a substantial risk for CIPN. These drugs include, but are not limited to platinum agents, taxanes, vinca alkaloids, proteasome inhibitors, immunomodulators and even new, targeted therapeutic agents. There are currently no approved treatments to prevent or treat CIPN, thus the neurotoxicity can be dose-limiting for some patients.^252^ Platinum drugs, particularly cisplatin and oxaliplatin, are an important component of numerous standard-of-care treatment regimens for pediatric and adult cancers; for example, oxaliplatin is a part of the FOLFIRINOX and FOLFOX protocols.

CIPN can persist after treatment is completed. Up to 40% of cancer patients continue to struggle with CIPN five years after treatment ends^252^—and 10% remain symptomatic after more than 20 years. Thus, CIPN directly affects cancer survivorship, quality of life, and may limit future treatment options if cancer recurs.^252^

**Fig. 5:**
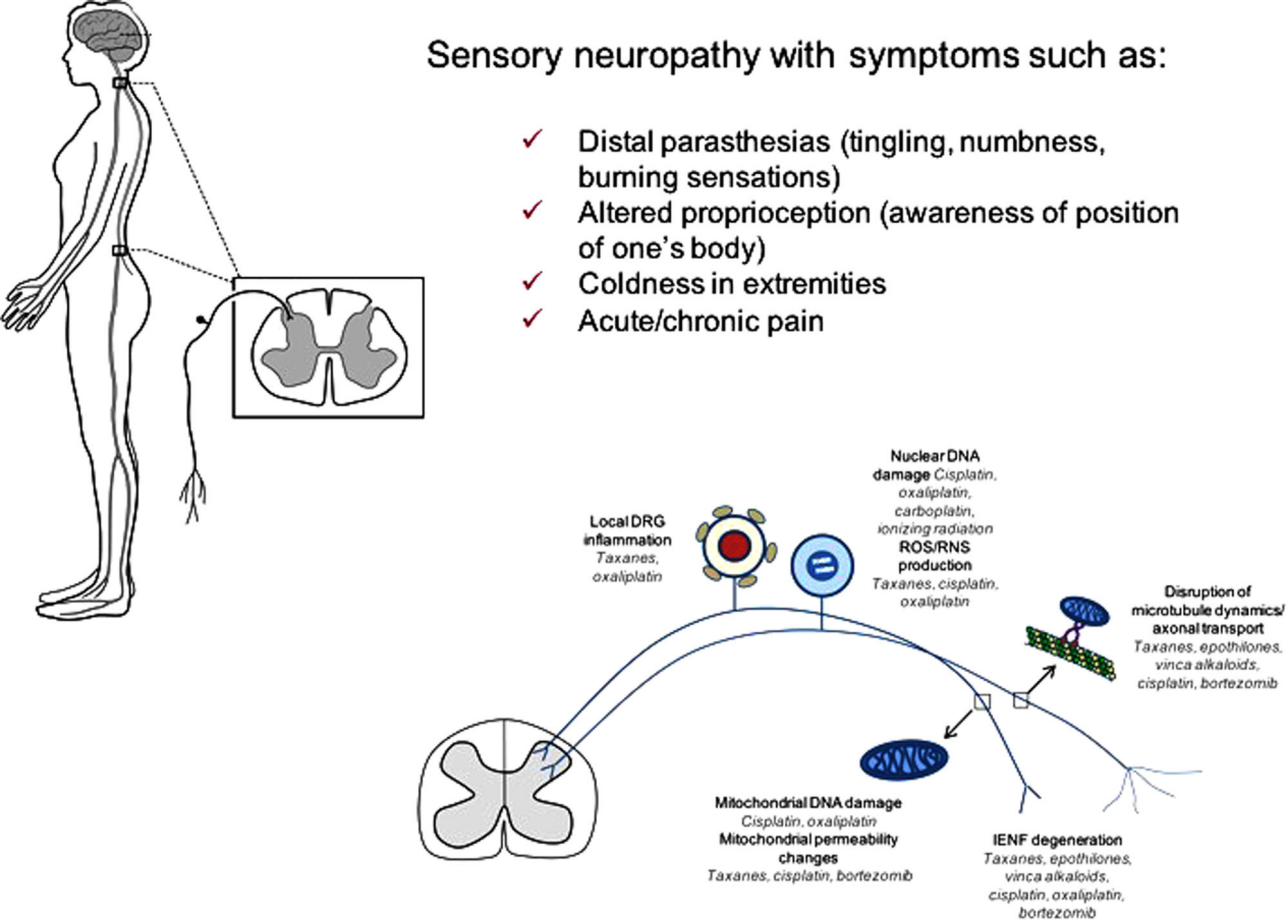
Chemotherapy-induced peripheral neuropathy (CIPN) produces significant DNA damage acted upon by Ref-1/APE1. Agents inducing oxidative DNA damage, such as cisplatin, oxaliplatin, ionizing radiation as well as other drugs are acted upon by the DNA BER pathway and specifically Ref-1/APE1. *ROS* reactive oxygen species, *RNS* reactive nitrogen species. DNA damage can occur on both nuclear and mitochondrial DNA in the dorsal root ganglion (DRG). Additional sites of action for some of the chemo-agents occurs in the axons. Inflammation has also been attributed to inducing oxidative DNA damage in DRGs. Patients report side effects, such as severe burning in fingertips, like putting fingers on hot stove, fingernails on a chalkboard, pain like needle stuck in toes, walking on hot coals, sandpaper at the bottom of feet, something crawling, blob of numbness, and feet are asleep. Parts of this figure are used by permission from previous figures after revision (see refs. 252, 278)

In previous studies using an experimental model of cultured sensory neurons, we established a causal relationship between CIPN and DNA damage and repair.^31, 106, 253, 254^ We demonstrated that reducing the activity of the DNA BER pathway by reducing expression of Ref-1/APE1 increased the neurotoxicity produced by anticancer treatment, whereas, augmenting the expression of Ref-1/APE1 lessened the neurotoxicity.^31, 255–257^ Additionally, we demonstrated that Ref-1/APE1’s DNA repair function—not the redox signaling function—is crucial for sensory neuron survival and function.^31^ We also demonstrated that the small-molecule redox inhibitor APX3330 protects sensory neurons from oxidative DNA damage caused by IR,^31^ cisplatin,^254^ and oxaliplatin (Fig. 5).

**Fig. 6:**
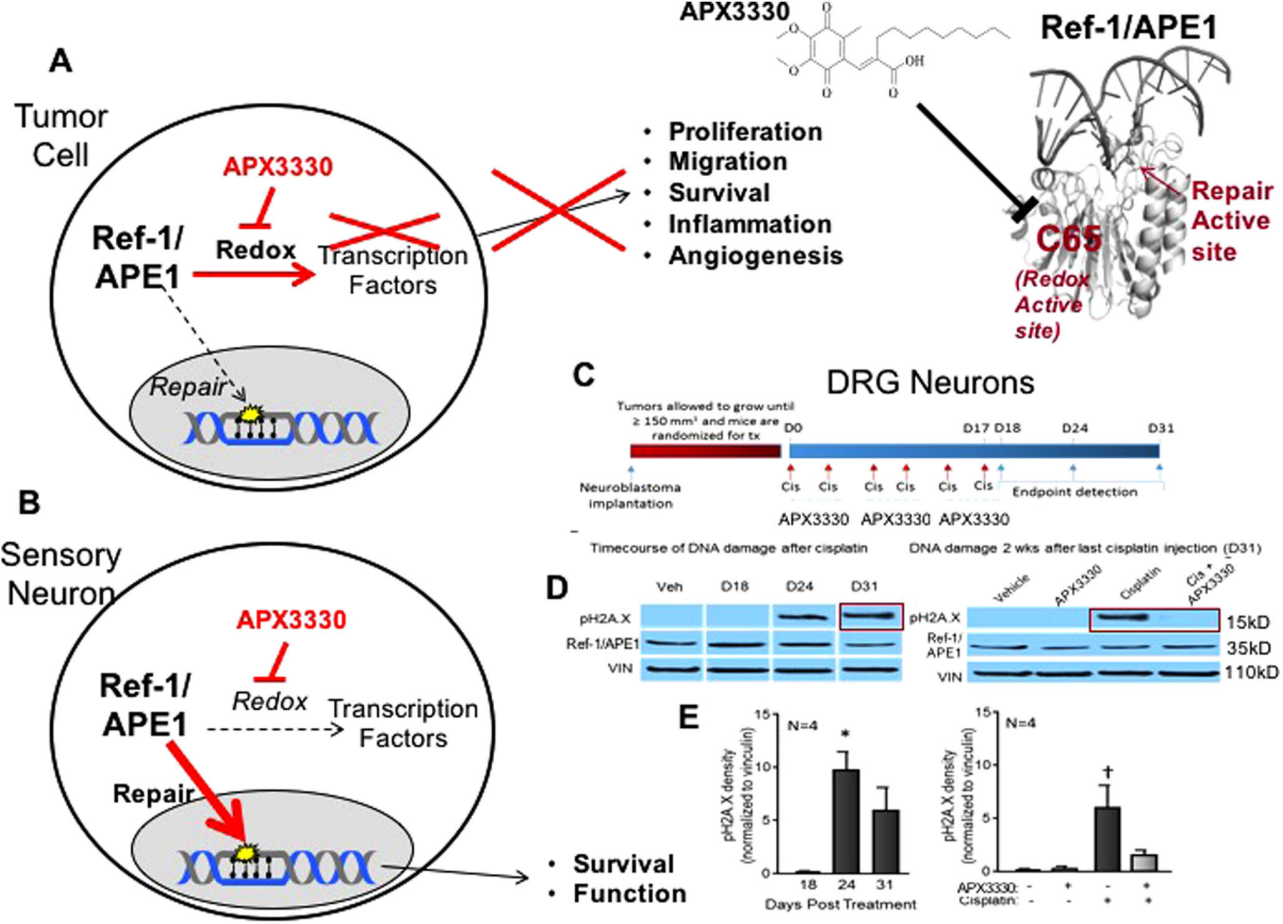
Differential role of Ref-1/APE1 redox inhibition in sensory neurons vs. tumor cells. **a** In tumor cells, Ref-1/APE1 redox inhibition as multiple downstream effects on tumor growth, survival, migration and tumor inflammation.^31, 106, 253, 254, 257^ **b** In sensory neuron cells such as DRG neurons, the addition of APX3330 does not have a negative effect on the cells, and promotes survival and functional protection through enhancement of Ref-1/APE1 DNA repair activity against oxidative DNA damaging agents (e.g., cisplatin, oxaliplatin) that invoked the DNA BER pathway. In the *lower right panel*, APX3330 attenuates neurotoxicity induced by systemic administration of cisplatin to tumor-bearing mice. **c** Treatment paradigm for investigation of the effects of cisplatin and APX3330 on DNA damage within DRG. Neuroblastoma cells were implanted subcutaneously into the right flanks of 6-week-old male NSG mice and allowed to proliferate until tumor volumes ≥150 mm^3^. Mice were then randomized for treatment with cisplatin ± APX3330 treatment. Cisplatin and APX3330 were administered concurrently for 3 weeks (Day 0–Day 17) and endpoints of neuronal toxicity were assessed within the DRG of mice at several time points following the last dose of cisplatin. **d** Representative blots demonstrating pH2A.X immunoreactivity at D24 and D31. **e** Quantification of pH2A.X immunoreactivity. An *asterisk* indicates statistical significance between D18 and D24 (**e**) as determined by a one-way ANOVA with Tukey’s post test with *p* < 0.05. A *cross* indicates statistical significance between Veh/Veh group and the Veh/Cis group (**e**) as determined by a two-way ANOVA with Bonferroni’s posttest with *p* < 0.05

This raises the question: how does a Ref-1/APE1 redox-specific inhibitor affect DNA repair activity? Although APX3330 is a targeted inhibitor of Ref-1/APE1’s redox function, it appears that, in the setting of sensory neurons, it can also enhance the protein’s DNA repair (AP endonuclease) activity (Fig. 6). Although this seems counterintuitive, on closer inspection it is not so unexpected. APX3330 causes the protein to unfold over time.^21, 22, 28^ This unfolding primarily alters the amino end of Ref-1/APE1, affecting its interactions with downstream transcription factor targets by perturbing the equilibrium of the protein’s folded/unfolded states and facilitating repair activity.^21, 28, 258^ This disengagement of Ref-1/APE1 from its Ref-1/APE1 redox activity could enhance Ref-1/APE1 repair endonuclease activity. When isolated sensory neurons are exposed to APX3330, a concentration-dependent increase in Ref-1/APE1 endonuclease activity occurs^254^—which is not observed in tumor cells.^29, 258, 259^ As discussed in previous paragraphs, we found that APX3330 protected sensory neurons from DNA damage and reactive oxygen species (ROS) production induced by agents such as IR, cisplatin and oxaliplatin.^31, 254^

A critical property of any putative therapeutic for neurotoxicity is that it will not compromise the anticancer function of the treatment(s) administered. Importantly, the enhancement of DNA repair activity by APX3330 was not observed in mitotic cells.^22, 258, 260^ We have shown that APX3330 negatively affects the growth and/or survival of tumor cell lines, patient-derived cell lines, and tumors in animal models.^13, 17, 18, 25, 27, 29^ Therefore, it is possible that APX3330 could protect postmitotic cells without altering the effects of anticancer drugs on tumor cells (Fig. 6). Additionally, APX3330 does not affect cisplatin or oxaliplatin’s tumor-killing efficacy in vivo, yet it protects DRG neurons from oxidative DNA damage (data unpublished). If further translational research further bears out these findings, APX3330 could be offered as a neuroprotective mechanism in humans, facilitating BER repair of oxidative DNA damage and protecting sensory neurons. In healthy cells, it appears that the DNA repair function—not the redox function of Ref-1/APE1—is necessary for sensory neuronal survival/function. That is opposite from tumor cells. Collectively, these data support the notion that APX3330 can be neuroprotective against cancer therapy without compromising treatment.

## Precision oncology- biomarkers

Biomarkers are at the frontier of precision oncology. Ideally, they provide diagnostic, prognostic, or pharmacologic information to inform patient care.^261^ Biomarkers may be genetic variants (polymorphisms), abnormal protein production/expression, or protein dysfunction unique to cancers.^261, 262^ The premise of pursuing Ref-1/APE1 as a potential biomarker is based on its contributions to both disease suppression and therapeutic agent resistance.^261^ Variants in DNA repair pathways are common; however, they may not alter protein synthesis.^261^ For example, Ref-1/APE1’s most frequently found variant, T1349G (a change in the 148 residue), does not seem to affect APE1 functionality. Further work with this variant in regards as to predictive biomarker use is ongoing.^262, 263^ Additionally, simulation analyses have predicted other SNPs, such as I64T or I64V (rs61730854; rs2307486) and P311S (rs1803120), in Ref-1/APE1 that could affect protein function and be potential biomarkers for treatment classification.^264^ Additional SNPs such as G39E (rs34632023) and Q51H (rs1048945) also have a predicted negative impact on Ref-1/APE1 DNA repair function.^264^

In searching for cancer-specific Ref-1/APE1 alterations possessing potential utility as a biomarker or a marker for patient selection criteria, two promising possibilities exist: overexpression and abnormal subcellular localization. Both are present in many cancers. In general, both overexpression and abnormal cytoplasmic-vs.-nuclear distribution of Ref-1/APE1 are associated with resistance to DNA-damaging agents, tumor aggressiveness, and poor prognosis.^3, 239, 261, 265, 266^

Ref-1/APE1 overexpression has been postulated to play a role in the increased ability of tumor cells to grow and metastasize as a result of increased gene expression of genes that are directed by numerous critical transcription factors regulated by Ref-1/APE1.^20^ As discussed in Section II, numerous cancers have altered levels of Ref-1/APE1 expression (also see Table 2, Fig. 4). Tumorigenesis, cancer aggressiveness, increased angiogenesis, radiotherapeutic and chemotherapeutic resistance, and overall poor prognosis are all associated with increased Ref-1/APE1 levels.^21, 159–163^ These cancers include prostate, colon, pancreatic, ovarian, non-small cell lung carcinoma, leukemias, MPNST, brain tumors, osteosarcoma, rhabdomyosarcoma, endothelial cell tumor, breast cancer (including triple-negative), hepatocellular carcinoma (HCC), head and neck, bladder, multiple myeloma, pediatric epdndymoma and retinoblastoma (Table 2).^3, 239, 261, 265, 267^ Additionally, low levels of Ref-1/APE1 appear to predict sensitivity to platinating agents for several cancers, especially NSCLC.^81, 263, 268^ Furthermore, a drop in serum Ref-1/APE1 levels pre- vs. post-treatment can indicate better overall survival or longer progression-free survival in some cancers, particularly NSCLC.^81^ Low Ref-1/APE1 levels confer radiosensitivity in pancreatic, colorectal, and cervical cancer cell lines.^178, 194, 269, 270^ Human HCC has elevated Ref-1/APE1 levels suggesting that over-expression in HCC correlates with cancer aggressiveness and indicates Ref-1/APE1 to be a promising marker.^160^ Additionally, increased Ref-1/APE1 cytoplasmic expression is a predictor of survival for HCC.^271^

Ref-1/APE1 normally localizes to the nucleus, with a much smaller but relevant localization to the mitochondria. A shift to greater cytoplasmic localization is present in some cancers; mounting evidence indicates that may be prognostic.^160, 191, 197, 271, 272^ The reason for this abnormal subcellular trafficking is yet unknown; perhaps post-translational modifications drive the protein’s redistribution or a general increase in Ref-1/APE1 levels result in a net cytoplasmic increase. APE1’s distribution pattern varies by cancer type and stage, the tumor’s genetic composition and microenvironment, and further work needs to be done in this area.

Currently the most promising work with Ref-1/APE1 as a biomarker is with regard to bladder cancer. This includes the development of an ELISA based Ref-1/APE1 assay. A 2016 study by Choi showed that an increase in Ref-1/APE1 expression at a cutoff of 0.376 ng/100μl was 82% sensitive and 80% specific for detecting bladder cancer in urine.^273^ A smaller study from 2015 showed higher sensitivity (90%) but lower specificity (59%) for utilizing Ref-1/APE1 in serum to detect bladder cancer.^274^ Quantitative, noninvasive measurement of Ref-1/APE1 expression in urine or serum may 1 day become a diagnostic biomarker for bladder cancer as well as other cancers with additional studies in this area.

## Bench to clinic

The effect of Ref-1/APE1 inhibition has not been tested in cancer clinical studies, however, Apexian Pharmaceuticals has recently received IND approval to conduct a clinical study of its Ref-1/APE1 inhibitor, APX3330. APX3330 is a novel, oral anticancer agent and the first drug to target Ref-1 for cancer. APX3330 was originally developed by Eisai (called E3330 by Eisai) as a Ref-1-NFkB signaling inhibitor for the treatment of inflammatory liver disease. Eisai evaluated APX3330 through comprehensive phase IIB development program in 422 patients, achieving positive efficacy results. The drug has not been approved in Japan nor the US which necessitates new oncology phase 1 trials. APX3330 is extremely well-tolerated at dose levels consistent with development as a cancer agent. It has IND and IRB approval. Eisai preclinical and clinical data demonstrates a safety profile that supports development of APX3330 for the treatment of various cancers. This includes (taken from the filed IND): (a) 13 week dog and rat studies with unremarkable safety findings, (b) Phase 1 (75 Japanese subjects) with single or multiple doses and Phase 2 (>350 Japanese subjects with hepatitis B or C; acute severe hepatitis or alcoholic hepatitis) with unremarkable safety findings, (c) No acute toxicity seen on neurologic, cardiovascular or pulmonary function, (d) Linear pharmacokinetics of APX3330 with little accumulation, (e) Absorption, distribution, metabolism and excretion of APX3330 understood, and (f) In Phase 2 trials, APX3330 was detected in serum at concentrations up to 147 μM; levels well above that required for anti-tumor effect in our models of pancreatic cancer. Eisai also concluded there were improvements in transaminase levels in patients with hepatitis B and C. APX3330 is well absorbed orally with a bioavailability of ≥60%. All of this data greatly de-risks the drug and due to its lack of toxicity profile, it will likely be easy to combine with other agents.

The phase 1, multicenter, open-label, dose-escalation oncology study will commence in 2017, with the goal of identifying a recommended phase 2 study dose to be used for subsequent development of the agent. The study population will include patients with recurrent or advanced cancer (i.e., solid tumors) for whom standard therapy offers no curative potential. APX3330 will be supplied as orally administered tablets and patients will receive a fixed dose of APX3330 twice daily (i.e., bid) each day of a 21-day cycle.

Additional study endpoints include pharmacokinetic and pharmacodynamic (PD) characterization of APX3330, the latter involving analysis of blood and tissue obtained from participants, including the following:

- Level of Ref-1/APE1 protein in whole blood using an ELISA assay
- RNA-sequencing to determine the effect of APX3330 on downstream transcription factors and their regulated genes involved in Ref-1/APE1 signaling, including, but not limited to HIF-1α, NFκB, STAT3, AP-1 and NRF2
- Analysis of CDA polymorphisms such as CXCL12 and CXCR4
- Effect of APX3330 treatment on cytokines and chemokines found in the blood
- Evidence of effect on (circulating tumor cells) CTCs after exposure to APX3330

**Fig. 7:**
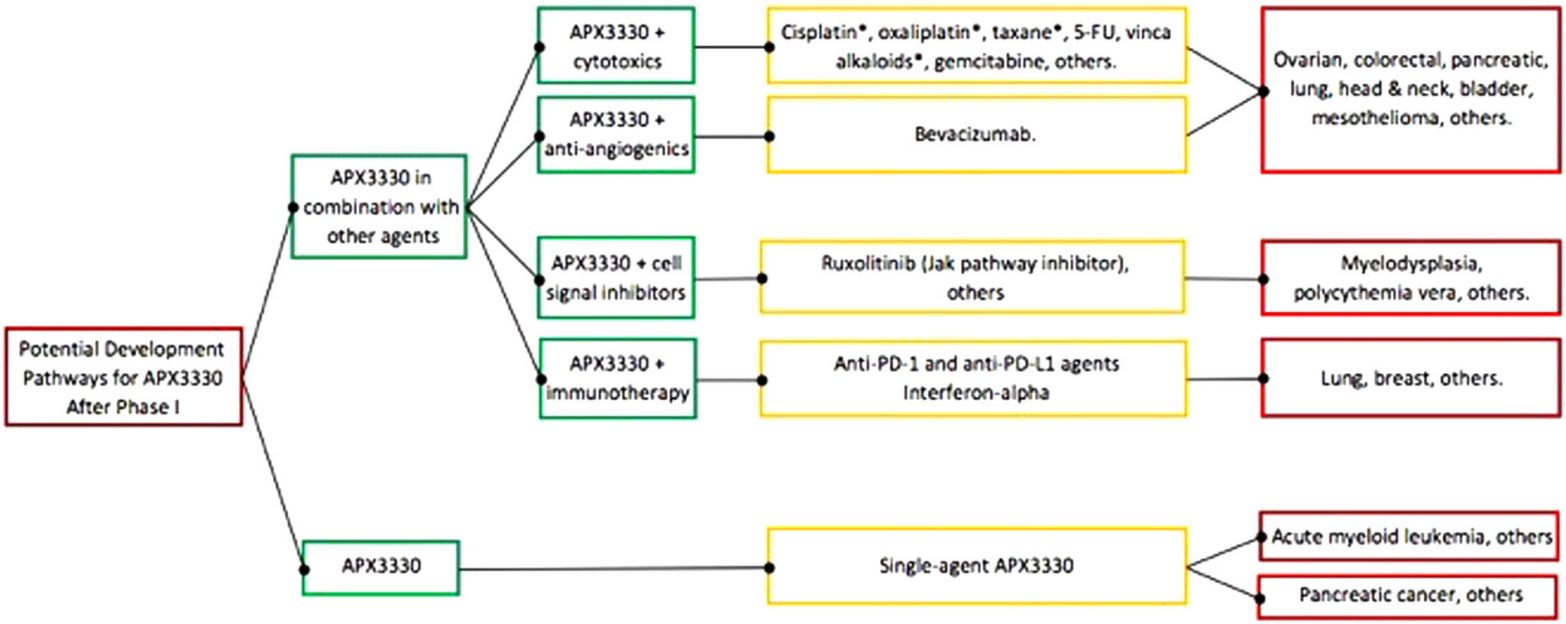
APX3330 has broad potential in a variety of cancers. Supportive pre-clinical data exists for APX3330 in combination with each drug listed in the diagram (*yellow boxes*) and for each indication (*red boxes*). The asterisk symbol indicates that in addition to anti-tumor activity, APX3330 provides neuroprotection when administered with agents causing oxidative damage to neurons. Definitive developmental plans await results of Phase I study and discussions with key opinion leaders

Phase 1 study results of single-agent APX3330 will inform the subsequent development of the agent, and pre-clinical data suggest a variety of potential pathways for clinical development of APX3330 whether alone or in combination with other anti-cancer agents. Figure 7 outlines these potential development pathways, each supported by existing preclinical data.

## Pipeline

Development of additional Ref-1/APE1 redox inhibitors based on APX3330 and related families of compounds is underway.^253^ A number of novel analogs have been synthesized based on structure–activity relationship. Changes include alterations of the dimethoxybenzoquinone with a napthoquinone ring, modification of the carboxylic acid, carbon chain on the double bond shortened, and substitution of the methyl group on the ring structure with hydrogen or various halogens.^29, 253, 259, 260^ APX3330 exists as a charged molecule at physiological pH; the addition of amide derivatives of carboxylic acid altered APX3330’s physical properties. Also, the lipophilic carbon chain was shortened on the double bond, making the new compounds less lipophilic^253^ [Patent 9,089,605]. These changes resulted in new compounds that exhibited greater potency than APX3330 during in vitro testing.^253^

The current compounds attack Ref-1/APE1 in a highly selective and specific manner, causing local unfolding of the protein and inhibiting its redox signaling function.^12, 21, 22, 28^ Identification of additional compounds outside of the chemical space of APX3330 and its analogs is not an easy task and is not readily amenable to high-throughput screening. Screening for molecules that block protein-protein interactions and, in this case, recognize an alternative redox-active conformation of Ref-1/APE1, are intrinsically complicated to find. One must rule out extraneous non-specific hits and target Ref-1/APE1 without unwanted toxicities or promiscuous activity.

## The future

Ref-1/APE1 continues to be an intriguing protein in its function and activities as well as a target for cancer therapeutics and role in other diseases. As discussed in this review, significant studies have identified the important interactions of Ref-1/APE1 with critical TFs in cancer as well as in other indications (Section II), confirming that Ref-1/APE1 is a key signaling node. Blocking the redox signaling function is a unique approach to alter tumor cell survival and growth and provides a novel approach to cancer therapeutics. Additionally, recent innovative studies using Ref-1/APE1 knockdown and single-cell RNAseq has identified unique, hypothesis-driven combinatorial approaches to partner the Ref-1/APE1 inhibitor APX3330 with FDA-approved drugs (Section III). This approach will allow specific pathways to be targeted via Ref-1/APE1 redox inhibition as well as uncover additional roles of Ref-1/APE1 in previously undiscovered signaling and mitochondrial pathways and metabolism. With APX3330 advancing to clinical trials, the role of Ref-1/APE1 in human cancer will be further elucidated.

In conclusion, there is significant interest in Ref-1/APE1 as a cancer target as well as potential use in other diseases. The advancement of the first clinical agent to target Ref-1/APE1 redox function in humans will offer insight into clinical uses, as will second-generation agents under development. With the identification of hypothesis-driven combinations of APX3330 and other FDA-approved drugs targeting selected pathways, a synthetic lethal approach for precision oncology will be forthcoming.

### Materials and methods

#### 3D Co-cultures

Patient-derived Pa03C tumor cells and CAF19 cancer-associated fibroblast cells were obtained from Dr. Anirban Maitra (Johns Hopkins) and cultured as previously described.^14, 17, 18, 180^ STR analysis (CellCheck with IDEXX BioResearch) was used to confirm the identity of the cells and that they were mycoplasma-free. Cells were passaged up to 10 times before new stocks were thawed. 3-dimensional tumor spheroids were cultured in DMEM containing 3% reduced growth factor matrigel (BD Biosciences) and 5% FBS as previously described.^14, 64, 275^ Cells were stably transduced as follows: tumor cells with TdTomato (red), CAF cells with EGFP (green).^64, 180^ 3D cultures were treated on Days 4 and 8 after plating with inhibitors as indicated and analyzed using Thermo ArrayScan high-content imaging system on Day 12 after plating.^276^

#### Western blot analysis

Western blots were performed as previously described^14, 17, 18, 25, 253^ with antibodies for Ref-1/APE1 (Novus Biologicals; Littleton, CO), phospho-STAT3 & total-STAT3 (Cell Signaling; Danvers, MA), phospho-histone H2AX (EMD Millipore; Billerica, MA), and Vinculin (Sigma; St. Louis, MO). All samples were processed and run in parallel.

#### Inhibitors

APX3330 was prepared and used as previously described.^14, 27, 28^ Ruxolitinib was dissolved in DMSO prior to dilution in media and use at the concentrations specified (Santa Cruz; Dallas, Texas).

#### In vivo CIPN studies in tumor bearing mice

The treatment protocol is presented in Fig. 6C. IMR32 Neuroblastoma cells^253^ were implanted subcutaneously into the right flanks of 6-wk old male NSG mice and allowed to proliferate until tumor volumes ≥ 150 mm^3^. Mice were then randomized for treatment with cisplatin ± E3330 treatment. Cisplatin and E3330 were administered concurrently for 3 weeks (Day 0–Day 17) and endpoints of neuronal toxicity were assessed within the DRG of mice at several time points following the last dose of cisplatin. pH2A.X immunoreactivity was performed on isolated DRG neurons as previously described^253, 254^ and shown in Fig. 6D. Quantification of pH2A.X immunoreactivity after normalization to vinculin was performed as previously described^253, 254^ (Fig. 6E). An asterisk indicates statistical significance between D18 and D24 as determined by a one-way ANOVA with Tukey’s posttest with p < 0.05. A cross indicates statistical significance between Veh/Veh group and the Veh/Cis group, as determined by a two-way ANOVA with Bonferroni’s posttest with p < 0.05. Mouse monoclonal anti–phospho-H2A histone X and vinculin antibodies were from EMD Millipore (Billerica, MA). Chemiluminescence secondary antibodies were from Roche Diagnostics (Indianapolis, IN). Ref-1 antibody was from Novus Biologicals, CO.^253, 254^ Cisplatin was purchased from Sigma-Aldrich (St. Paul, MN). Neuroblastoma cells, IMR32, were obtained from American Type Culture Collection (Manassas, VA) and grown in RPMI 1640 medium supplemented with 10% fetal bovine serum. Cell line identity was confirmed by DNA fingerprint analysis (IDEXX BioResearch, Columbia, MO) for species and baseline short-tandem repeat analysis testing. All cell lines were 100% human and a nine-marker short tandem repeat analysis is on file. They were also confirmed to be mycoplasma free. All samples were processed and run in parallel.

### Data availability

The data discussed in this review are available from the cited publications. Additional data supporting studies presented or discussed are available on request from Dr. Mark R. Kelley.
